# Pelvic organ prolapse: Women’s experiences of Accessing Care & Recommendations for improvement

**DOI:** 10.1186/s12905-023-02832-z

**Published:** 2023-12-18

**Authors:** Louise Carroll, Cliona O’. Sullivan, Catherine Doody, Carla Perrotta, Brona M. Fullen

**Affiliations:** 1 University College Dublin School of Public Health, Physiotherapy and Sports Science, Dublin, Ireland; 2 University College Dublin Centre for Translational Pain Research, Dublin, Ireland; 3 Tipperary University Hospital, Clonmel, Co. Tipperary, Clonmel, Ireland

**Keywords:** Pelvic organ prolapse, Physiotherapy, Women's health, Central sensitization, Pelvic floor exercises, Treatment

## Abstract

Up to 50% of women will develop pelvic organ prolapse (POP) over their lifetime. Symptoms include pain, bulge, urinary, bowel and sexual symptoms affecting all aspects of a woman’s life.

Many women with POP symptoms present initially to primary care settings. Research has shown these interactions are often unsatisfactory, with women reporting their health care professional (HCP) trivialized their symptoms or appeared to have poor knowledge about pelvic floor dysfunction (PFD).

**Aim** The aim of this qualitative study was to explore experiences of younger women seeking treatment for POP and their recommendations for improvements.

**Methods** Ethics approval was obtained (LS-21-01-Carroll-Ful). Women with POP were recruited from an online support group (*n* = 930 members). Inclusion criteria: adult women, diagnosed with POP and aware of their POP stage. Following informed consent, a demographic questionnaire, interview questions and the Central Sensitization Inventory (CSI) were forwarded. Semi-structured zoom audio-recorded interviews were conducted. Thematic analysis was undertaken; transcripts coded, and themes identified.

**Results** Fourteen women aged 32–41, parity 1–3, with POP Grade 1–3 participated. Many women reported HCPs as dismissive or not appreciative of the impact of their condition. Others described interactions with HCPs who they felt listened, understood the impact of their POP, gave simple explanations, a positive prognosis and outlined a realistic treatment plan.

Current antenatal education, post-partum care and primary HCP screening for PFD were identified by women as deficient. Many highlighted delays in accessing specialist care for POP. Women made several recommendations for improvements to the current model of care.

**Conclusions** Increased focus on person-centred care, particularly emotional support, information and education may improve younger women’s experiences when seeking care for POP.

## Introduction

Female Pelvic organ prolapse (POP) is defined by the International Urogynecological Association (IUGA) and International Continence Society (ICS) as a departure from normal sensation, structure, or function, experienced by a woman in reference to the position of her pelvic organs [1].

Prevalence rates worldwide are between 9 to 20% and higher prevalence when POP is defined by vaginal examination rather than symptoms [2].

Symptoms of POP include a sensation of a bulge in the vagina, pelvic pressure, feeling “like a tampon is falling out,” groin or low back pain, urinary or faecal incontinence, difficult bowel movements, sexual dysfunction and lack of vaginal sensation [3]. These symptoms can have significant impact on women and their lived experience has been explored as part of the current study, but reported elsewhere [4].

POP is less common in women under the age of 45 than in older women. The limited research to date would suggest that this cohort differs from women who develop POP at a later stage of life in that younger women with POP have a higher incidence of first degree relatives with the condition [5] and are more likely to experience higher symptom bother that may plateau or decrease at an age corresponding to menopause despite increasing anatomic POP [6].

Pelvic organ prolapse can be treated by expectant (watchful waiting), conservative or surgical management. Two thirds of women initially opt for conservative management (Kapoor et al.) [7]; local oestrogen application [8], vaginal pessaries, pelvic floor exercises, lifestyle interventions (including weight loss, treating constipation, avoiding straining to empty the bowel and heavy lifting) [9]. Type of surgery used generally depends on patient characteristics and preferences, POP compartment and surgical skill available [10–12].

Eight international guidelines or recommendations for POP have been established and compared by Tsiapakidou et al. (2021) [13]. Four recommendations for POP diagnosis were described across all guidelines; assessment of POP, taking a detailed personal history and physical examination, objective assessment of POP using the POP quantification system (POP-Q), and consideration of imaging studies. Regarding conservative management two recommendations were common to all guidelines: pelvic floor muscle training and use of vaginal pessaries. In terms of surgical management all guidelines recommended detailed patient counselling, treating only symptomatic cases of POP, consideration of apical fixation during surgical correction and use of biological or synthetic implants in recurrent POP.

There is little research into healthcare professional (HCP) awareness or use of clinical practice guidelines for management of POP; however one study has shown that half of specialist pelvic health physiotherapists treating women with POP reported having no guidelines outlining treatments that should be offered for POP [14]. Other research has identified that healthcare practitioners (including medical students and HCPs in primary, secondary and tertiary care) underestimate, under-screen and may be unfamiliar with treatment for common pelvic floor dysfunctions (PFDs). Knowledge deficits in several areas have been observed, including screening, assessment, treatment, and timely referral to specialist care [15–18].

Research has shown that the majority of primary HPCs reported never or hardly ever screening female patients for POP [19]. Among HCPs working in a tertiary care centre, fewer than half of doctors reported being confident undertaking a pelvic floor assessment while the majority (86%) of staff (including midwives, doctors and gynaecological nurses) had not heard of the Oxford Grading for pelvic floor muscle strength [19].

Prentice et al. [15] observed that fewer than half of women presenting with PFD to their primary HCP in the USA would have treatment initiated within a year of symptom onset. Less than 70% of medical students expected to be comfortable with pessary fitting and management for POP and UI at the time of graduation [20] and 48% of primary care providers would refer patients with POP to specialist care immediately as opposed to initiating treatment themselves [15].

As with all research the patient’s voice in healthcare is important. The current study examines the experiences of these women seeking treatment for POP and also their recommendations for optimal management.

## Methods

Methods were previously described in detail [4]. A qualitative research methodology was employed using the EQUATOR standards for reporting research [21]. An interview guide, informed by the contemporary literature, the principal investigator’s (PI) expertise and the study aims was developed and piloted. The interview guide focused on (i) the lived experience of POP, reported separately [4] and (ii) women’s experiences of seeking treatment for POP and their recommendations for optimal management. Ethics approval was obtained from University College Dublin’s Human Research Ethics Committee (LS-21-01-Carroll-Ful). Women with POP registered to an online support group (*n* = 930 members) were invited to participate by an advertisement on the support group website outlining the study.

Women interested in participating were invited to contact the PI through details on the advertisement. The PI screened them for eligibility via zoom, explained the study and forwarded an information leaflet and interview guide via e-mail. The women were given a period of 1 week to consider participating. They were then contacted by the PI and any questions they had were answered. Once the electronic informed consent form was received, they were accepted into the study.

Validated patient-reported outcome measures (Central Sensitization Inventory (CSI) [22] and Pelvic Organ Prolapse Quality of Life Questionnaire (P-QOL) [23, 24] were completed by participants. Semi-structured zoom recorded interviews were conducted at any time of the day or day of the week convenient to participants, in their homes during Covid 19 lockdowns. Thematic analysis was undertaken and themes and subthemes defined. The current paper reports on women’s experiences of seeking treatment and their recommendations for improvements to POP management.

## Results

Semi-structured interviews were conducted with 14 women diagnosed with POP. The length of the interviews ranged from 40 to 100 minutes.

### Participant demographics

Participants were female, aged between 32 and 41 years (mean age of 36.79 ± 3.3 years) and mean parity 2 ± 0.5. Women with a range of POP stages participated: Stage I (*n* = 5), Stage II (*n* = 6) with Stage III (*n* = 3) women. Five women reported having episiotomy. Seven (50%) had a history of instrumental (vacuum/forceps) birth with one reporting a history of severe perineal trauma (SPT) (3rd degree tear). Participant demographics including co-morbidities are summarised in Table 1 (below). Further demographic details relating to participants scores for the Central Sensitisation Index (CSI) [22] and Pelvic Organ Prolapse Quality of Life (P-QOL) [23] are reported elsewhere [4]. In summary, 87% (*n* = 12) of participants had central sensitisation (CS) as per the Central Sensitisation Index with 57% (*n* = 8) above the cut-off score of > 40 (more severe CS). In addition, symptom impact was reported in all domains of the Pelvic Organ Prolapse Quality of Life (P-QOL) questionnaire [22, 23].

**Table 1:** Participant Demographics

Participant No.	Age Years	Births (Number)	Birth Type	POP Type & Stage (dominant compartment)	Other Pelvic Floor Symptoms	Comorbidities
1	40	2	1st birth: forceps, epis, 3rd degree tear 2nd birth: c-section	Stage I anterior wall	SUI	Neck Injury, Migraine, Tension headaches
2	38	2	Vaginal, epis on both	Stage I anterior wall	Urinary Frequency, Dyspareunia	
3	41	2	Vaginal	Stage III posterior wall	SUI	IBS, Anxiety, Panic attacks
4^b^	39	2	Vaginal, IOL, forceps	Stage III anterior & apical	Recurrent UTIs	
5	39	2	1st birth: vacuum, forceps, epis 2nd birth: SVD	Stage I unsure of involved compartment		Migraine, Tension Headaches
6	35	2	Vaginal	Stage II anterior wall	Incomplete bladder emptying	
7	38	3	1st birth: IOL, forceps, epis Subsequent 2 births: vaginal	Stage I anterior, posterior & apical	Pelvic pain	IBS
8	37	2	Vaginal	Stage III posterior wall	Bowel urgency with running	
9	39	2	Vaginal	Stage II anterior wall	Urinary urgency	
10^a^	33	2	Vaginal	Stage II anterior wall	SUI	Interstitial cystitis, TMJ disorder
11^a^	32	2	1st birth: vacuum, epis 2nd birth: vaginal	Stage I posterior wall	Difficulty emptying bowel	Autoimmune condition, IBS
12	36	3	Vaginal	Stage II anterior wall	SUI	IBS
13	34	1	Vacuum	Stage III anterior wall		
14^a^	41	2	1st birth: vacuum 2nd birth: elective c-section	Stage II anterior and posterior wall	Difficulty emptying bowel	Breast Cancer, Depression

epis = Episiotomy, IOL = Induction of Labour, SVD=Spontaneous Vaginal Delivery, SUI=Stress Urinary Incontinence, UTI=Urinary Tract Infection. ^a^pessary use ^b^pessary use (unsuccessful)

## Interview themes

Two core themes (Current Care and Optimal Care Recommendations) with a number of subthemes were identified from the interviews and are discussed in detail below.

### Current care theme

This theme had five subthemes; (i) Knowledge (ii) Barriers to accessing care (iii) Healthcare professionals (iv) Antenatal & postnatal care (vi) Limited treatment options and the juxtaposition of women’s and men’s health.

#### Knowledge

Most women reported either never having heard of POP or believing that it was a condition that affected older people.

*“I didn’t even know what she meant until my stepmother had a prolapse, but she was in her 70s. I had to Google it see what it was, and I was like, oh yeah that’s what the doctor told me might happen to me.” T1P12L23.*

*“You kind of just think it’s older women it happens to, like I’d never have thought that my bowel would be bulging into my vaginal wall. I didn’t know that was even possible.” T3P21L16.*

Others described feeling symptoms and being unable to identify the cause, believing that certain symptoms were normal to experience after giving birth or not being educated prior to giving birth that POP could happen.

*“I didn’t know what it would it feel like, or I didn’t know what it would entail, or what the recovery would be like.” T5P13L26.*

*“I’d been so ignorant to it; I’d heard the word prolapse...probably, in passing. I can’t remember in those pre appointments where...you go to the classes...I don’t remember the word prolapse being mentioned.” T3P8L6.*

On receiving a diagnosis of POP, many of the women reported receiving little additional information.

*“My GP told me nothing…she was very nice and compassionate but… perhaps in her defence she did seem to genuinely think I would get an appointment, within a few weeks. So maybe that’s why…she didn’t give me any information.” T1P12L5.*

*“The GP gave me no information about how to relieve the symptoms…actually it was the group on the internet that said put the pillow underneath your hips… and heat, and that will help relieve the symptoms.” T5P13L23.*

Others received information they perceived as worrying or giving little hope of improvement.

*“The consultant’s view was that things are only going to get worse through menopause.” T3P2L10.*

*“Sometimes it felt like it was being over-practical and there was no hope.” T10P14L24.*

Some of the women felt that the information or advice they received was impractical.

*“The physio would encourage, you know, sit for an hour, stand for an hour, lie down for an hour and that’s just not possible.” T4P4L11.*

*“She’s like do not stop it, you need to do more, you need to use weights*. *Which I… just two small children, I’m not getting to do it very regularly.” T7P2L20.*

As a result most of the women reported searching other sources for information and that much of the knowledge they gained about POP was self-sourced, mainly through social media, internet searches and family.

*“The information I got, it was kind of self-sourced initially.” T2P11L32.*

*“I’ve learned more for myself than I got from health professionals.” T9P9L18.*

*“I got more information from social media than I did from anything else.” T8P10L5.*

#### Barriers to accessing care

Initial diagnosis of their POP was often delayed for women. This was due to women’s own lack of knowledge regarding normal post-natal pelvic floor function, a general societal acceptance of women’s bodies being ‘damaged’ after childbirth and difficulty bringing up the topic with their healthcare provider.

*“When you’ve never had a baby before, you have no idea what’s normal after.” T15P27L23.*

*“And isn’t it funny like there’s probably plenty of men with incontinence but it’s always a woman in the ad, you’d never see a man like happy out, running, playing soccer in a nappy.” T8P18L33.*

*“It wasn’t really asked and I wasn’t going to be like, hey do you want to look at me? Like it wasn’t going to be like that.” T7P10L51.*

After initial diagnosis, women reported long delays in gaining access to specialised care for their POP; this was due to long waiting lists for services, covid 19 related delays and the cost barrier to accessing private care.

*“Then I had to wait weeks and weeks for the physio.” T5P2L29”.*

*“She sent me to the gynaecologist, but that referral because of covid… took more than a year.” T14P1L29.*

*“An awful lot of these services aren’t available very easily through the public health system. So if you haven’t got the resources to pay privately for…a women’s physio appointment, you know, an awful lot of women aren’t going to get that, are they?” T15P29L31.*

#### Healthcare professionals

Having overcome the barriers to seeking care for their POP, many women encountered HCPs (in both primary and secondary care) who they perceived as being dismissive or not appreciating the impact of their condition.

*“I went to a gynae, and it was like, you know it’s grand it’s just prolapse, like it’s just… really common.” T4P7L12.*

*“A lot of things you might ask him that you knew were kind of important, he would be like yeah, that’s not, that doesn’t really matter. And you’d be like, no, I think it does.” T15P25L31.*

*“He told me, you’ve stage one of everything, you’re grand come back to me in 20 years for surgery, no context with it whatsoever.” T8P2L10.*

In some cases, women felt that their HCP lacked knowledge about POP.

*“I didn’t think they were particularly well versed in…correct and good information on prolapse.” T14P26L17.*

*“I just get the impression it’s not something, many of them are educated in, or have come across.” T1P21L23.*

In terms of interactions between women and their HCPs during consultations in relation to their POP diagnosis, many of the women reported receiving very little or worrying information, a hopeless prognosis or being given advice to significantly restrict many activities, including general physical activity. This has previously been discussed [4].

*“The GP told me don’t lift anything heavy, don’t lift up your kids.” T5P12L18.*

*“The physio said, definitely don’t be walking too much, because you’re doing quite well and if you aggravate that, it could actually get worse.” T10P8L2.*

*“The consultant’s view was that things are only going to get worse, through menopause.” T3P2L10.*

Others reported very positive interactions and appreciated being able to access knowledgeable HCPs who they felt listened, understood the impact of their POP, gave explanations which they understood, a positive prognosis and outlined a realistic treatment plan. Specialist physiotherapists tended to be the HCPs most often perceived by women as being knowledgeable about POP, taking time to explain the condition and its treatment and giving a more positive prognosis.

*“I found the physio who I had was very… understood the major impact that it was having on my life and trying to understand it.” T4P10L23.*

*“The information that she gave me was really quite clear. It was delivered in a way that made me feel reassured that it wasn’t sort of life ending and going to take over everything.” T12P10L27.*

*“She was so clear and so comforting. She just made everything so easy to understand… the information was brilliant… She showed me on a model what she was going to do first and she said she’d be talking me through all of it, and she was just really helpful.” T14P6L43.*

Women reported that this type of interaction fostered feelings of hope, empowerment and self-efficacy.

*“She made me feel a lot more kind of positive about it. Like that I could do something myself, I suppose she kind of made me feel more empowered to fix it myself.” T13P2L24.*

*“She’d maybe give me three exercises and they might be slightly harder than the previous time. And I would usually leave her room… kind of like going ‘I’m not able to do them’. But I suppose it was motivation then. It was like, right, have to have it mastered before you go back to her.” T10P10L22.*

*“No matter how bad I felt going into her, she always was able to give me some little bit of hope.” T15P25L5.*

#### Antenatal & Postnatal Care

Most women described receiving little or no information in antenatal classes regarding risks of common birth interventions, normal pelvic floor function and signs and symptoms of pelvic floor dysfunction both prior to and after birth.

*“I felt like there was probably little to no mention of the risks that could occur in labour.” T3P14L54.*

*“Like you have baby preparing classes about how do you look after the baby. But we never talk about how to look after yourself post natally.” T8P13L22.*

*“People talk about the importance of pelvic floors, but they never say why.” T8P12L15.*

*“I don’t remember it ever being mentioned in my antenatals.” T12P11L25.*

Several of the women noted a lack of informed consent during the birth process, particularly in the context of forceps birth.

*“I wish my doctor had said, these are the options that we might use during birth, and these are the risks associated with them. You know, I had no idea that forceps could cause any damage.* “*T1P13L18.*

*“Why didn’t somebody say…why didn’t you bring me for a section, rather than, you know, doing three attempts of a forceps, why didn’t you use the vacuum instead?” T4P11L6.*

*“I wasn’t given a choice, I mean I probably could have spoke up and maybe they would have not done it, but … I don’t know, it wasn’t like anyone said, do you… are you okay with us using forceps?” T1P15L34.*

Post-natal care was almost universally highlighted among the women as inadequate, with many describing post-natal check-ups as rushed, cursory or mostly focused on their baby.

*“I was just sort of, annoyed that it was a thing of like, how crap the post-natal system is that I hadn’t been checked.” T5P3L13.*

*“This six week GP business is a bloody joke, like, you know an awful lot of GPs don’t examine a woman…as I said to you an awful lot of them know next to nothing about prolapse and have even less interest.” T15P29L16.*

*“When I had the check-up after the second baby, there wasn’t time for my check-up, we were both on the same appointment, so they basically looked, assessed the baby.” T9P115L32.*

#### Limited treatment choices & the juxtaposition of women’s and men’s health

Some women highlighted the limited treatment options for women with POP.

*“There isn’t a lot happening as far as I know, in terms of treatment.” (T15P34L32).*

*“I’d love if someone said okay if you do this it’ll be gone, or if you have surgery, it’d go away but like you know…you can know that the guarantees of that is very limited and the treatment plans are very limited.” T8P9L17.*

Others compared attitudes to women’s health issues to that of men’s health.

*“If that was a man, they’d be saying…what are you doing putting up with that…get yourself to the doctor, surely there’s a tablet or some treatment or something… Yet we’re telling women ‘no sure it’s grand, just piss yourself there away now, just put these black nappy things on you, they’re nice and sexy’, you know?” T15P30L3.*

*“I just think if you had men having penis problems, it would be a lot different.” T8P15L36.*

*“You know, it’s just the reality of men versus women, they’re just, it’s just there’s no appreciation, I guess of the… medical conditions and issues that women have.” T3P18L3.*

### Optimal care recommendations theme

This theme also consisted of three subthemes; (i) Information (ii) Services (iii) Healthcare Professionals.

#### Information

Receiving information about all types of pelvic health and dysfunction was seen as important by all participants. Several key areas of information were highlighted, including general information, more information on birth interventions, what to expect during post-partum recovery, signs and symptoms of possible pelvic dysfunction, where and how to seek help for pelvic dysfunction and tips on managing the symptoms of POP once diagnosed.

Women also described when this information might be given. Routine post-partum healthcare interactions were highlighted by most women as an ideal time to give information on pelvic dysfunction, particularly POP.

Verbally screening for pelvic health issues post-partum was highlighted by women as a simple, quick intervention for HCPs to identify patients who might require specialist follow-up to ensure return to normal pelvic floor function.

*“Even to just… to ask the questions of, how you’re feeling would be helpful…because it didn’t even occur to me to say it, because I was like, sure I just had a 4.2 kilo baby, of course I’m sore.” T7P10L45.*

*“I think it should be, it should be part and parcel of just an appointment…going, how’s your pelvic floor, are you leaking, are you suffering any incontinence, do you notice anything, do you feel internally okay, do you notice anything that doesn’t feel right? They should be asking those questions.” T4P12L35.*

Women identified GPs, practice nurses and/or smear takers as the HCPs they would encounter most frequently throughout the various life stages. They suggested a number of ways that pelvic floor screening could be incorporated into routine healthcare throughout a woman’s life span. These included HCPs routinely enquiring about the symptoms of pelvic dysfunction when women attend for other reasons, screening for POP during smear tests, placing printed information material in their surgeries and performing an adequate physical examination when concerns are raised.

*“You go to the GP and you’d say you know, you go, go for a sore throat or whatever. And then they turn around and say, oh and how is your prolapse? Any changes in that?” T3P22L11.*

*I know it’s often an issue in menopause but I don’t know, I suppose, if people are going for menopause symptoms it’s worth mentioning, that like, it’s something to be aware of. T7P11L28.*

*Even if it’s just a leaflet on a wall that it’s in people’s radar of something to be aware of, maybe. T7P11L40.*

*“Just continue to have that that conversation with women in whatever way that that can be and I guess like a smear test would probably be the most annual or tri-annual thing that you would continue always.” T11P15L10.*

Many of the women also noted that pelvic health information could be included in school sexual health or personal development curricula.

*“If you’re wanting to help tackle that kind of wider societal issue of women’s health not being valued and not being looked after, not being important… I think probably sexual health classes in school are a very good foundation way to start, so you could be teaching young girls about pelvic floor exercises and I’m sure it could be part of whatever type of curriculum they do in terms of sexual health.” T15P31L3.*

#### Services

##### Pelvic health screening

All participants identified pelvic health screening at routine healthcare visits as important.

*“Even to just… to ask the questions of, how you’re feeling would be helpful…because it didn’t even occur to me to say it, because I was like, sure I just had a 4.2 kilo baby, of course I’m sore.” T7P10L45.*

*“I think they should ask you, ‘I see you had a vacuum or forceps; how has that recovery been, have you felt this, have you felt that?’” T15P31L34.*

##### Updating of antenatal classes

Most of the women highlighted antenatal and postnatal care as areas requiring improvement, suggesting that antenatal care could be improved by updating antenatal classes. They stressed the importance of including information on general pelvic health, normal post-partum recovery and signs and symptoms that might indicate pelvic dysfunction. They also emphasised the need for more information on birth interventions and their implications for future pelvic health to facilitate fully informed consent during childbirth.

*“Even for five minutes… this is an area that you need to look after…you should be doing your pelvic floor exercises and if you have any concerns afterwards you should be making sure to get checked out and go to your GP or a women’s health physio.” T10P20L29.*

*“It would have been nice to talk about…the risks if you have a ventouse or a vacuum delivery. And if you have forceps you know you’re at risk of prolapse.” T8P11L32.*

*“… let people know that it can happen and it’s not that bad, like it can be fixed.” T14P7L1.*

*“If we’re going to prepare women for having babies, we need to be preparing them for their aftercare as well.” T15P27L15.*

##### Routine post-partum pelvic health assessment

Being made aware of the existence of pelvic health physiotherapists and how to access this care was also emphasised as crucial to women in order to receive timely assessment and treatment. Many of the women were of the opinion that routine post-partum physiotherapy assessment and/or treatment should be the norm as part of a holistic and woman-centred maternity service.

*“My friends have said, in France, like you get like free women’s health physio if you have a kid. I feel like that, that’s the kind of support that should be offered, you know, whether it’s free or not, but it should be offered at least that.” T2P14L5.*

*“There’s no women’s physio included in your post-natal, which there should be. You’re not even shown your... most women aren’t even doing their kegel exercises properly and they don’t even know that they’re not.” T5P3L24.*

##### Timely referral to specialist pelvic health services

Early referral, assessment and treatment by specialized, well-trained and experienced healthcare professionals was seen as important for women with POP.

*“You really need to see a specialist because it is kind of a niche area.” T1P21L33.*

*“I don’t know how easy that is in Ireland with the HSE but you know, getting people the right support as quickly as possible.” T2P17L16.*

*“I would expect people to say, like this is your pathway out of this. So, I’ll refer you to a women’s physio… and it will probably take this long to heal, you’ll probably have to do exercises, don’t do this, don’t do that and go to a women’s physio.” T5P15L24.*

*“A well trained physio, do you know? Like there is obviously differences to ones just out of college - like you want to have, you know, a specialized physio would be the ones you’d be looking at.” T8P16L11.*

Women also highlighted the importance of peer support, which they felt may be useful for helping to manage symptoms and reduce feelings of isolation.

*“Like other people that you know are kind of the same stage of life, as you or whatever you know? And you read their stories and they sound so personal or something, you’re kind of like...oh yeah. I don’t know, it’s...it’s quite supportive.” T13P20L5.*

*“I think the Facebook group was obviously brilliant in terms of getting information and kind of knowing and being able to ask a question and I find that actually (it sounds terrible) but like, asking women who’ve experienced stuff.” T2P12L50.*

#### Healthcare professionals

##### Better communication, early referral, more information

Women recognised that not all the HCPs they encountered would or should have specialist training in the area of women’s or pelvic health. However, they emphasised HCP-patient communication as an important area for improvement, allowing HCPs to hear women’s pelvic health concerns, give basic information and assess or refer to specialist care as appropriate which may facilitate more timely access to the relevant services.

*“They need to be willing to engage in these conversations and refer people properly and if they don’t know anything about it, you know, either find out or refer the person on to somebody who does.” T15P33L15.*

*“I would expect people to say, like this is your pathway out of this. So, I’ll refer you to a women’s physio, just go to a women’s physio and it will probably take this long to heal, you’ll probably have to do exercises, don’t do this, don’t do that and go to a women’s physio.” T5P15L24.*

Women valued HCPs taking time to discuss and give information on activities that are ‘safe’ and those that may be likely to worsen POP, how to manage their POP symptoms, how POP is treated and what their POP prognosis might be.

*“I wish my GP had said… when she diagnosed… I wish she could have given me, at least some cursory info. That like, if she could have said, okay, maybe hold off on doing X, Y, Z until you are able to see the gynaecologist or a physio.” T1P18L21.*

*“It would be nice if you got, you know, kind of a full explanation of the rationale behind different opinions, like, I know I would have had conversations with my gynaecologist about, kind of surgery options.” T15P34L33.*

## Discussion

The current study reports on younger women’s experiences of seeking care for their condition and their recommendations for improvements to the current model of care for POP. Two themes emerged with associated subthemes; current care (knowledge, barriers to accessing care, healthcare professionals, antenatal and postnatal care and limited treatment choices and the juxtaposition of women’s and men’s health) and optimal care (information, services and healthcare professionals).

Whilst some subthemes under the current care theme reflect the literature, exploration of women’s opinions of current management and recommendations for optimal care for PFD or POP has been limited to only one other study to date [25].

In keeping with what has previously been well documented in the literature [26–29], women reported having low levels of knowledge about POP and other pelvic floor problems. They described being given little information at diagnosis and as a result self-sourcing information, usually from the internet or social media. This allowed on many occasions for patients to become experts on their condition, with women describing sometimes feeling they knew more about POP than the HCPs they were consulting.

Women described being unaware that POP could happen, especially in younger women and many felt that their antenatal education did little to prepare them for the reality of post-partum recovery. Burmann et al’s research [30], published as far back as 2012, as well as more recent publications reflect this, reporting women’s shame, shock and feelings of unpreparedness for pain and pelvic floor problems experienced post-partum [25, 31].

This lack of information in antenatal preparation for birth is surprising, given that up to 75% of women giving birth vaginally will experience some type of pelvic floor trauma with up to 29% reporting POP symptoms post-partum [32–34]. Other research has suggested that encouraging women to perform pelvic floor muscle training (PFMT) during pregnancy may significantly shorten duration of labour and reduce the rate of severe perineal trauma (third and fourth degree tears) [35].

Antenatal classes are designed to prepare women for childbirth [36]. However, women suggested there was a need to update them to include information on birth interventions as well as what to expect during post-partum recovery, PFMT, return to exercise, symptoms of PFD to look out for and information on where to get help if required as important elements of comprehensive pelvic floor education antenatally.

They suggested that specialist physiotherapists, as experts in pelvic health and PFD would be ideally positioned as the HCP to contribute this information. There is a lack of data regarding physiotherapist’s current level of involvement in the delivery of antenatal classes worldwide and also the optimal time to communicate the required information.

National guidelines or standards for antenatal class content (Australian Competency Standards for CBE, National Standards for Antenatal Education in Ireland; internationally NICE and WHO guidelines on antenatal care) include some general recommendations for topics for inclusion in classes [37–40]. Of note, none of these include information on pelvic health and post-partum information as proposed by women in the current study; however, the NICE guideline on antenatal care NG201 does recommend information on post-natal self-care be given to women after 28 weeks of pregnancy [39].

One study found that standardizing antenatal classes had a greater effect on reducing caesarean section rates than antenatal classes of variable quality and content, suggesting that developing and standardizing other class content (such as that focused on pelvic health and post-partum recovery) may be the optimal way to approach inclusion of this information [41].

None of the women who had forceps assisted birth recalled receiving information on the well-documented risks of pelvic floor dysfunction [32, 42–44] either antenatally or in the birth room when their consent was sought. This raises the question of whether consent to certain procedures was fully informed in some cases and is an important consideration for both HCPs delivering antenatal education and those caring for women and their babies during pregnancy and birth.

Similar concerns have previously been raised, with Woolery warning that “although there are instances where courts disregard the informed consent doctrine in the light of the use of forceps…these decisions should not be relied on as judicial permission for ignoring informed consent.” Similarly, Dietz et al. has highlighted informed consent for forceps birth as a particularly urgent issue given that forceps carries a high risk of morbidity for mothers and babies. O′ Boyle et al. also highlighted informed consent as an important part of daily obstetric practice, in the context of significant risk of pelvic floor injury associated with certain birth interventions [45–47]. The literature concurs with the findings of the current study that women want to be provided with more information about potential complications of vaginal birth and the risk of future PFD attached to birth interventions [30, 46, 48, 49].

All of the women felt that post-natal care could be improved by including routine post-partum screening for pelvic floor disorders and instruction on PFMT. They also suggested information-giving and screening to be carried out by primary HCPs at routine healthcare visits such as GP visits, smear testing or community nursing visits. Currently there is a dearth of evidence regarding preventative post-partum PFMT and its effect on POP development [50]; however some research has shown promising results for prevention and treatment of post-partum stress incontinence [34, 51], quality of life (QOL), sexual function [52], pelvic floor muscle function and satisfaction rates [53].

There is ample evidence that pelvic floor muscle training reduces pelvic floor symptoms associated with POP [54–60], while most other conservative treatment requires further research to confirm its effectiveness [49, 61, 62]. Despite this, HCPs often fail to give adequate guidance with PFMT and rarely evaluate its correct performance [29].

Our study group had high levels of sensitization, which has previously been described in the literature. Vij et al. (2019) found a 32% prevalence of central sensitization in a wide age range of women with POP [63]. Other research suggests that in younger women with mild POP, severe symptom bother may be more associated with pelvic floor myofascial pain (which has been associated with central sensitization) than with anatomic prolapse [64–66]. Patients with central sensitization have been shown to have lower satisfaction with healthcare encounters, perhaps suggesting a different aetiology for their POP symptoms which is not being adequately addressed by current assessment and management approaches [67]. Some countries provide free or subsidised pregnancy and/or post-partum pelvic floor rehabilitation programmes for all post-partum women [68, 69]. Specific instruction on correct technique with PFMT and more comprehensive post-partum follow-up has been highlighted as desirable by women in other research [30, 69]; however because of limited exploration of women’s opinions and the benefits of preventative interventions for PFD, what form this might take is unclear.

The need for timely referral to specialist physiotherapists and or secondary or tertiary care was highlighted by all participants as a crucial aspect of optimal care for POP. This reflects the literature, where long delays in referral for both POP and other lower urinary tract symptoms have been noted in women of all ages in primary and secondary care [17]. It also highlights the need for awareness and training of HCPs in identification of PFDs, as well as clear pathways of referral and appropriate services to manage these conditions.

Barriers for seeking care for PFD among the women reflect findings in the literature to date, however there has been little examination of women’s experiences of receiving care [25, 29].

Many women with POP symptoms tend to present initially to primary care settings [14]. In the current study, when women sought care for their POP symptoms HCPs, particularly those encountered in primary care, were often perceived as being dismissive of their concerns and uninformed about POP. HCP communication was also an area identified by women as problematic, often with little or no explanation of POP received at diagnosis and vague or impractical advice given. This is consistent with other research, which found women’s complaints were trivialised, opportunities for early diagnosis and referral were missed and information given was inconsistent and unclear [25, 31].

In addition, HCPs themselves have been demonstrated to often have insufficient levels of knowledge or to be uncomfortable with managing POP [16, 18, 19, 70]. It has been shown that HCPs do not tend to routinely screen or provide support for PFD, perhaps due to this discomfort with the subject [19, 20, 71–73]. Even in tertiary care, pelvic floor examination method has been shown to vary in quality and in some cases HCPs are not aware of basic criteria of standardised pelvic floor assessment [19].

Pelvic health physiotherapists were generally reported as being knowledgeable about POP and spending time explaining the condition and it’s treatment in a way that women could understand. This has previously been described in the research and may be due to more contact time with patients and additional training and specialisation in the area than other primary HCPs [25]. Current research endorses the role of specialist physiotherapists in primary care [72, 73], particularly given the barriers to effective management of pelvic floor dysfunction including POP by other primary HCPs [71] discussed above which were also highlighted by women in the current study. There is a need for enhancing undergraduate education in women’s health. Research involving medical students in the US in 2009 noted an imbalance between learning opportunities related to advanced topics in obstetrics and gynaecology and more common women’s health conditions, such as PFD. It argued that as qualified clinicians PFD will be commonly encountered and that as such, opportunities to educate future physicians about PFD are being missed [74].

Similarly, half of midwifery students surveyed in the UK and Spain reported missing teaching in several key areas relating to pelvic health [75]. Even for those qualified, research has indicated that in the absence of adequate training and or standardized guidance regarding carrying out PFMT, registered midwives may not feel confident to teach or educate women about it [76].

Although the utilisation of specialist pelvic health physiotherapists in primary care has been shown to be cost effective, accessible, acceptable to patients and efficacious, many physiotherapy undergraduate programmes include little or no formal teaching in the area of PFD and pelvic floor examination [77, 78]. As a result physiotherapists graduate with variable levels of introductory knowledge of the pelvic floor and PFD. In most European countries, competence in pelvic health physiotherapy is gained through post graduate education, but there are at present no defined standards of competence to practice as a pelvic health physiotherapist [77, 78].

In Ireland, access to specialist pelvic health physiotherapy through the public health system is by GP or consultant gynaecology referral. This leads to long waits for physiotherapy assessment and treatment and may present another barrier to women seeking care for POP and other PFD, particularly for those women from lower income households who cannot afford to access private care. The formation of new ambulatory gynaecology hubs currently underway nationwide will still require GP referral, but may speed up access to specialist physiotherapy services and decrease unnecessary GP and consultant visits. These hubs have been shown to be a highly acceptable approach in a sample of Irish patients presenting for treatment of gynaecological conditions [79]. Defined basic standards of competence in the area of PFD and pelvic floor examination, and clear pathways of care are required for all primary HCPs treating women with PFD. This would allow adequate assessment, referral and/or treatment and information-giving. Prescription, demonstration and evaluation of PFMT is a simple evidence-based intervention that could be carried out by GPs and practice or public health nurses as well as pelvic health physiotherapists. The communication of basic information regarding POP and instruction in how to perform PFMT is valued by women and increases self-efficacy and hope [25, 30, 80].

Women need access to impartial, evidence-based information regarding their POP and both conservative and surgical treatment options. This information should ideally be given both verbally at the time of consultation with their HCP and in print or other formats. Women also require time to consider their options and discuss further with their HCP if necessary.

## Strengths

Our study had several strengths. The use of qualitative research methodologies facilitated the richness and in-depth information gained from study participants.

This study explores a problem that has not been well researched specifically in the younger age group and provides direction for the education of women and healthcare professionals and the development of services that meet the needs of younger women with POP.

## Limitations

Women were recruited from an online social media peer support group, having actively sought further information and support with their condition. This may imply a level of access to and knowledge of technology as well as self-efficacy and health literacy which may not be reflective of all women with POP.

Half of the women in the sample had instrumental birth and this is a higher rate than in the general population. The findings that women desire more information regarding birth interventions may be reflective of high levels of birth interventions experienced in this sample.

A high prevalence rate of central sensitisation was found among participants in this study. This has previously been shown to be associated with negative experiences with healthcare providers, and potentially could contribute to reported dissatisfaction with HCP encounters.

## Conclusions

This study elucidated the challenges faced by women with POP in diagnosis and management of their condition. Women recommend several cost-effective solutions including updating existing antenatal classes and verbal screening for PFDs particularly POP, at routine healthcare visits. They also suggest an additional service of routine post-partum check-ups with the aim of prevention and early diagnosis of POP.

## Data Availability

All relevant data is contained within the manuscript.

## References

[1] Haylen BT, De Ridder D, Freeman RM, Swift SE, Berghmans B, Lee J, at al. (2010). An international Urogynecological association (IUGA)/international continence society (ICS) joint report on the terminology for female pelvic floor dysfunction. Neurourology and Urodynamics: official journal of the international continence. Society..

[2] Barber MD, Maher C (2013). Epidemiology and outcome assessment of pelvic organ prolapse. Int Urogynecol J..

[3] Roos AM, Thakar R, Sultan AH, Burger CW, Paulus AT (2014). Pelvic floor dysfunction: women’s sexual concerns unraveled. J Sex Med..

[4] Carroll L, O’Sullivan C, Doody C, Perrotta C, Fullen B. Pelvic organ prolapse: The lived experience. Plos one. 2022;17(11):e0276788. 10.1371/journal.pone.0276788 PMC9629641 36322592

[5] Alcalay M, Stav K, Eisenberg VH (2015). Family history associated with pelvic organ prolapse in young women. Int Urogynecol J..

[6] Miedel A, Tegerstedt G, Maehle-Schmidt M, Nyrén O, Hammarström M (2008). Symptoms and pelvic support defects in specific compartments. Obstet Gynecol..

[7] Kapoor DS, Thakar R, Sultan AH, Oliver R (2009). Conservative versus surgical management of prolapse: what dictates patient choice?. Int Urogynecol J..

[8] Weber MA, Kleijn MH, Langendam M, Limpens J, Heineman MJ, Roovers JP (2015). Local oestrogen for pelvic floor disorders: a systematic review. PLoS One..

[9] Abrams P, Andersson KE, Apostolidis A, Birder L, Bliss D, Brubaker L, Cardozo L, Castro-Diaz D, O'connell PR, Cottenden A, Cotterill N (2018). 6th international consultation on incontinence. Recommendations of the international scientific committee: evaluation and treatment of urinary incontinence, pelvic organ prolapse and faecal incontinence. Neurourol Urodyn..

[10] Raju R, Linder BJ (2021). Evaluation and management of pelvic organ prolapse. Mayo Clinic proceedings.

[11] Gray TG, Giarenis I (2021). Surgical management of pelvic organ prolapse. Obstet Gynaecol Reprod Med..

[12] Ko KJ, Lee KS (2019). Current surgical management of pelvic organ prolapse: strategies for the improvement of surgical outcomes. Investig Clin Urol..

[13] Tsiapakidou S, Nygaard CC, Falconi G, Pape J, Betschart C, Doumouchtsis SK, CHORUS: An International Collaboration for Harmonising Outcomes, Research and Standards in Urogynaecology and Women's Health (i-chorus. org) (2021). Systematic review and appraisal of clinical practice guidelines on pelvic organ prolapse using the AGREE II tool. Neurourol Urodyn..

[14] Hagen S, Stark D, Dougall I (2016). A survey of prolapse practice in UK women’s health physiotherapists: what has changed in the last decade?. Int Urogynecol J..

[15] Prentice A, Bazzi AA, Aslam MF (2019). Treatment patterns of primary care physicians vs specialists prior to subspecialty urogynaecology referral for women suffering from pelvic floor disorders. World J Methodol..

[16] Madsen AM, Hickman LC, Propst K (2021). Recognition and Management of Pelvic Floor Disorders in pregnancy and the postpartum period. Obstet Gynecol Clin N Am..

[17] Wong JW, Kaneshiro BE, Oyama IA (2019). Primary care physician perceptions of female pelvic floor disorders. Hawai'i J Med Public Health..

[18] Krissi H, Eitan R, Edward R, Peled Y (2012). Diagnostic delay in secondary care for lower urinary tract and pelvic organ prolapse symptoms in women. Arch Gynecol Obstet..

[19] Mazloomdoost D, Crisp CC, Kleeman SD, Pauls RN (2018). Primary care providers’ experience, management, and referral patterns regarding pelvic floor disorders: a national survey. Int Urogynecol J..

[20] Kandadai P, Mcvay S, Larrieux JR, O'Dell K (2016). Knowledge and comfort with pessary use: a survey of US obstetrics and gynecology residents. Female Pelvic Med Reconstruct Surg..

[21] O’Brien BC, Harris IB, Beckman TJ, Reed DA, Cook DA (2014). Standards for reporting qualitative research: a synthesis of recommendations. Acad Med..

[22] Mayer TG, Neblett R, Cohen H, Howard KJ, Choi YH, Williams MJ, Perez Y, Gatchel RJ (2012). The development and psychometric validation of the central sensitization inventory. Pain Practice..

[23] Digesu GA, Khullar V, Cardozo L, Robinson D, Salvatore S (2005). P-QOL: a validated questionnaire to assess the symptoms and quality of life of women with urogenital prolapse. Int Urogynecol J..

[24] Neblett R, Cohen H, Choi Y, Hartzell MM, Williams M, Mayer TG, Gatchel RJ (2013). The central sensitization inventory (CSI): establishing clinically significant values for identifying central sensitivity syndromes in an outpatient chronic pain sample. J Pain..

[25] Abhyankar P, Uny I, Semple K, Wane S, Hagen S (2019). Women’s experiences of receiving care for pelvic organ prolapse: a qualitative study. BMC Womens Health..

[26] Liu J, Tan SQ, Han HC (2019). Knowledge of pelvic floor disorder in pregnancy. Int Urogynecol J..

[27] Good MM, Korbly N, Kassis NC, Richardson ML, Book NM, Yip S, Saguan D, Gross C, Evans J, Harvie HS, Sung V (2013). Prolapse-related knowledge and attitudes toward the uterus in women with pelvic organ prolapse symptoms. Am J Obstet Gynecol..

[28] O’Neill AT, Hockey J, O’Brien P, Williams A, Morris TP, Khan T, Hardwick E, Yoong W (2017). Knowledge of pelvic floor problems: a study of third trimester, primiparous women. Int Urogynecol J..

[29] Chen CCG, Cox JT, Yuan C, Thomaier L, Dutta S (2019). Knowledge of pelvic floor disorders in women seeking primary care: a cross-sectional study. BMC Fam Pract..

[30] Buurman MB, Lagro-Janssen AL (2013). Women’s perception of postpartum pelvic floor dysfunction and their help-seeking behaviour: a qualitative interview study. Scand J Caring Sci..

[31] Mirskaya M, Lindgren EC, Carlsson IM (2019). Online reported women’s experiences of symptomatic pelvic organ prolapse after vaginal birth. BMC Womens Health..

[32] Blomquist JL, Carroll M, Muñoz A, Handa VL (2020). Pelvic floor muscle strength and the incidence of pelvic floor disorders after vaginal and cesarean delivery. Am J Obstet Gynecol..

[33] Tennfjord MK, Engh ME, Bø K (2020). The influence of early exercise postpartum on pelvic floor muscle function and prevalence of pelvic floor dysfunction 12 months postpartum. Phys Ther..

[34] Sigurdardottir T, Bø K, Steingrimsdottir T, Halldorsson TI, Aspelund T, Geirsson RT (2021). Cross-sectional study of early postpartum pelvic floor dysfunction and related bother in primiparous women 6–10 weeks postpartum. Int Urogynecol J..

[35] Sobhgol SS, Smith CA, Dahlen HG (2020). The effect of antenatal pelvic floor muscle exercises on labour and birth outcomes: a systematic review and meta-analysis. Int Urogynecol J..

[36] Jones C, Wadephul F, Jomeen J (2019). Maternal and paternal expectations of antenatal education across the transition to parenthood. Br J Midwifery..

[37] Australian Competency Standards for Childbirth and Parenting Education https://capea.org.au/wp-content/uploads/2021/01/National-Competency-Standards-2019-inpageversion.pdf

[38] Irish National Standards for Antenatal Education https://www.hse.ie/eng/about/who/healthwellbeing/our-priority-programmes/child-health-and-wellbeing/antenatal-ed.pdf

[39] Nice Guideline NG201 Antenatal Care https://www.nice.org.uk/guidance/ng201/resources/antenatal-care-pdf-66143709695941

[40] Abalos E, Chamillard M, Diaz V, Tuncalp Ӧ, Gülmezoglu AM (2016). Antenatal care for healthy pregnant women: a mapping of interventions from existing guidelines to inform the development of new WHO guidance on antenatal care. BJOG Int J Obstet Gynaecol..

[41] Cantone D, Lombardi A, Assunto DA, Piccolo M, Rizzo N, Pelullo CP, Attena F. A standardized antenatal class reduces the rate of cesarean section in southern Italy: A retrospective cohort study. Medicine. 2018;97(16) 10.1097/MD.0000000000010456 PMC5916688 29668615

[42] Handa VL, Blomquist JL, McDermott KC, Friedman S, Muñoz A (2012). Pelvic floor disorders after childbirth: effect of episiotomy, perineal laceration, and operative birth. Obstet Gynecol..

[43] Urbankova I, Grohregin K, Hanacek J, Krcmar M, Feyereisl J, Deprest J, Krofta L (2019). The effect of the first vaginal birth on pelvic floor anatomy and dysfunction. Int Urogynecol J..

[44] de Leeuw JW, Daly JO (2021). Forceps and vacuum: one goal, two entities. Int Urogynecol J..

[45] Woolery W (2000). Informed consent issues throughout the birthing process. J Legal Med..

[46] Dietz HP, Caudwell Hall J, Weeg N (2021). Antenatal and intrapartum consent: implications of the NSW consent manual 2020. Aust N Z J Obstet Gynaecol..

[47] O'Boyle AL, Davis GD, Calhoun BC (2002). Informed consent and birth: protecting the pelvic floor and ourselves. Am J Obstet Gynecol..

[48] Wilson D, Dornan J, Milsom I, Freeman R (2014). UR-CHOICE: can we provide mothers-to-be with information about the risk of future pelvic floor dysfunction?. Int Urogynecol J..

[49] Bugge C, Strachan H, Pringle S, Hagen S, Cheyne H, Wilson D (2022). Should pregnant women know their individual risk of future pelvic floor dysfunction? A qualitative study. BMC Pregnancy Childbirth..

[50] Wu JM, Vaughan CP, Goode PS, Redden DT, Burgio KL, Richter HE (2014). Prevalence and trends of symptomatic pelvic floor disorders in US women. Obstetrics Gynecol..

[51] Mørkved S, Bø K (2014). Effect of pelvic floor muscle training during pregnancy and after childbirth on prevention and treatment of urinary incontinence: a systematic review. Br J Sports Med..

[52] Hadizadeh-Talasaz Z, Khadivzadeh T, Ebrahimipour H, Khadem Ghaebi N (2019). Explaining facilitators and barriers to treatment-seeking in women with pelvic organ prolapse: a qualitative study. Iran J Obstet Gynecol Infertil..

[53] Gagnon LH, Boucher J, Robert M (2016). Impact of pelvic floor muscle training in the postpartum period. Int Urogynecol J..

[54] Hagen S, Glazener C, McClurg D, Macarthur C, Elders A, Herbison P, Wilson D, Toozs-Hobson P, Hemming C, Hay-Smith J, Collins M (2017). Pelvic floor muscle training for secondary prevention of pelvic organ prolapse (PREVPROL): a multicentre randomised controlled trial. Lancet..

[55] Hagen S, Stark D. Conservative prevention and management of pelvic organ prolapse in women. Cochrane Database Syst Rev. 2011;12 10.1002/14651858.CD003882.pub4 PMC12621084 22161382

[56] Panman CM, Wiegersma M, Kollen BJ, Berger MY, Lisman-Van Leeuwen Y, Vermeulen KM, Dekker JH (2017). Two-year effects and cost-effectiveness of pelvic floor muscle training in mild pelvic organ prolapse: a randomised controlled trial in primary care. BJOG Int J Obstet Gynaecol..

[57] Alves FK, Riccetto C, Adami DB, Marques J, Pereira LC, Palma P, Botelho S (2015). A pelvic floor muscle training program in postmenopausal women: a randomized controlled trial. Maturitas..

[58] Brækken IH, Majida M, Engh ME, Bø K (2015). Can pelvic floor muscle training improve sexual function in women with pelvic organ prolapse? A randomized controlled trial. J Sex Med..

[59] Li C, Gong Y, Wang B (2016). The efficacy of pelvic floor muscle training for pelvic organ prolapse: a systematic review and meta-analysis. Int Urogynecol J..

[60] Wang T, Wen Z, Li M (2022). The effect of pelvic floor muscle training for women with pelvic organ prolapse: a meta-analysis. Int Urogynecol J..

[61] Adams EJ, Thomson AJ, Maher C, Hagen S. Mechanical devices for pelvic organ prolapse in women. Cochrane Database Syst Rev. 2004;2 10.1002/14651858.CD004010.pub2 15106231

[62] Choi KH, Hong JY (2014). Management of pelvic organ prolapse. Korean J Urol..

[63] Vij M, Davies A, Dua A, Freeman R (2019). The proportion of women with central sensitivity syndrome in gynecology outpatient clinics (GOPDs). Int Urogynecol J..

[64] Dixon AM, Fitzgerald CM, Brincat C (2019). Severity and bother of prolapse symptoms in women with pelvic floor myofascial pain. Int Urogynecol J..

[65] Meister MR, Sutcliffe S, Badu A, Ghetti C, Lowder JL (2019). Pelvic floor myofascial pain severity and pelvic floor disorder symptom bother: is there a correlation?. Am J Obstet Gynecol..

[66] Vandyken B, Keizer A, Vandyken C, Macedo LG, Kuspinar A, Dufour S (2021). Pelvic floor muscle tenderness on digital palpation among women: convergent validity with central sensitization. Braz J Phys Ther..

[67] Brook G (2020). Postpartum physiotherapy: a commentary on evidence-based guidance and current practice, including a survey of International Organization of Physical Therapists in Women’s health delegates. J Pelvic Obstetric Gynaecol Physiother..

[68] Boissonnault J, Kuhn A, Meeker M, Strong S, Stephenson RG (2020). An international survey of women's and pelvic health physical therapy organizational practice. J Women's Health Phys Ther..

[69] Herron-Marx S, Williams A, Hicks C (2007). AQ methodology study of women's experience of enduring postnatal perineal and pelvic floor morbidity. Midwifery..

[70] Huang WC, Lau HH, Su TH (2022). Minimal requirement in urogynecological knowledge for obstetrics and gynecology specialists. Taiwa J Obstetr Gynecol..

[71] Ghijselings L, Pauwaert K, Verla W, Beeckman D, Van de Putte D, Pattyn P, Everaert K (2021). Primary care providers’ knowledge about the therapeutic management of refractory pelvic floor dysfunctions in Flanders, Belgium: a cross-sectional study. Acta Chir Belg..

[72] Mazloomdoost D, Crisp CC, Kleemaan SD, Pauls RN (2018). Primary care providers experience, management and referral patterns regarding pelvic floor disorders: a National Survey. Int Urogynecol J..

[73] Dufour S, Hondronicols A, Flanigan K (2018). Conservative primary Care of Urinary Incontinence and Pelvic Organ Prolapse in primary health care. Ann Reprod Med Treat..

[74] Mueller ER, Kenton K, Rogers RG, Fenner DE (2009). Are we missing an opportunity to teach future physicians about female pelvic floor disorders?. Int Urogynecol J..

[75] Webb SS, Skene ER, Manresa M, Percy EK, Freeman RM, Tincello DG (2021). Evaluation of midwifery pelvic floor education and training across the UK and Spain. Eur J Obstetr Gynecol Reproduct Biol..

[76] Terry R, Jarvie R, Hay-Smith J, Salmon V, Pearson M, Boddy K, MacArthur C, Dean S (2020). “Are you doing your pelvic floor?” an ethnographic exploration of the interaction between women and midwives about pelvic floor muscle exercises (PFME) during pregnancy. Midwifery..

[77] Frawley HC, Neumann P, Delany C (2019). An argument for competency-based training in pelvic floor physiotherapy practice. Physiother Theory Practice..

[78] McPherson K, Nahon I, Waddington G (2022). Entry level women’s health physiotherapy curricula in Australia. Eur J Phys..

[79] Uzochukwu I, Burke C, Ni Bhuinneain M. Patient satisfaction and acceptability: a journey through an ambulatory gynaecology clinic in the West of Ireland. Irish Medical Journal. 27814437

[80] Lawson S, Sacks A (2018). Pelvic floor physical therapy and women's health promotion. J Midwifery Womens Health..

